# The Role of Nitric Oxide on Male and Female Reproduction

**DOI:** 10.21315/mjms2022.29.2.3

**Published:** 2022-04-21

**Authors:** Sulagna DUTTA, Pallav SENGUPTA

**Affiliations:** 1 Faculty of Dentistry, MAHSA University, Selangor, Malaysia; 2 School of Medical Sciences, Bharath Institute of Health Sciences and Research (BIHER), Chennai, India; 3 Faculty of Medicine, Bioscience and Nursing, MAHSA University, Selangor, Malaysia

**Keywords:** erectile dysfunction, fertilization, infertility, nitric oxide, oxidative stress, reactive nitrogen species

## Abstract

Nitric oxide (NO), a reactive nitrogen species, is a molecule of high physiological as well as pathological importance. Physiological mechanisms mediated by NO mainly include angiogenesis, growth, puberty and senescence. NO has vital roles in normal reproduction, including steroidogenesis, gametogenesis and the regulation of germ-cell apoptosis. In females, NO stimulates an inflammatory cascade to induce ovulation, decreases steroidogenesis in luteal and granulosa cells, and acts as a paracrine factor to mediate reproductive cycles and implantation. In males, NO is a key player for steroidogenesis, erectile functions, sperm capacitation and acrosome reaction. Moreover, NO is also a regulator of Sertoli cell-germ cell interaction and maintenance of the blood-testis barrier. In pathological conditions such as infections, increased nitric oxide synthase (NOS) activities stimulate the excessive synthesis of NO which acts as a proinflammatory mediator inducing oxidative stress (OS), which is detrimental to reproductive functions in both males and females. During impregnation, the overproduction of NO results in uterine epithelial cell inflammation and immune rejection of implantation. Excessive NO synthesis disrupts gonadal functions, and induces germ cell apoptosis and oxidative damage to the germ cells. This review elucidates how the differences in NO expression levels account for its beneficial and adverse impacts upon male and female fertility.

## Introduction

Nitric oxide (NO) has a brief half-time (approximately a couple of seconds) and possesses different potentials for biochemistry and physiology. First discovered in 1978, this molecule was nominated as the molecule of the year in 1992 (1–2). It is an internal and intracellular cell messenger that plays an important role in maintaining the body homeostasis (2). By synthesising cyclic guanosine monophosphate, NO generally accomplishes its function. The synthesis of the NO from L-arginine is mediated by the NO synthase (NOS). This enzyme exists in three isoforms: i) nervous; ii) endothelial and iii) induction (1). In the different tissues, NO cascade stimulates various pathways, e.g. they emerge as a vasodilator factor and a known relaxing factor in the cardiovascular system originating from endothelium (2). NO is nevertheless regarded as a neurotransmitter in the nervous system. It is also involved in neutrophil-induced toxicity of cells, platelet aggregation, blood flow, synaptic transmission and long-term memory loss in some cases (3–4). In addition to the aforementioned functions, NO is involved in ovulation, menstruation, and sperm capacity and motility (5–6). NO is an essential paracrine messenger which, in the elementary and endocrine organs, participates in several physiological and pathophysiological events (7). In addition, NO has certain immune system functions including antiviral and antimicrobial effects, immune system excitation or suppression, and cytoprotection (8).

## Physiological Sources of Nitric Oxide

Three NOS isoforms including neural NOS (nNOS), endothelial NOS (eNoS) and inducible NOS (iNOS) can be used to produce NOs in mammals. Mitochondrial NOS, located exclusively in mitochondria, were discovered recently (9). In addition, iNOS and eNOS are found in various reproductive tissues, such as the granulosa, theca and oocyte cytoplasm. The majority of apoptotic cascades contributes to reactive nitrogen and oxygen species (9).

## Mechanisms of Nitric Oxide Biosynthesis

L-arginine, molecular oxygen and reduced phosphate nicotinamide-adenine-dinucleotide (NADPH) as co-substrate are used by all NOS isoforms. In the amino-terminal oxygenase domain, a functional NOS transfers electron from NADPH to the heme (4, 10). The electron on the hem site is used for reducing and activating oxygen (O_2_) and oxidising L-arginine into L-citrulline and NO (11). The NOS enzyme must take two main steps in order to synthesise NO, including hydroxylated l-arginine into n-hydroxy-l-arginine and oxidising l-citrulline and NO into n-hydroxy-l-arginine (12–14). Binding to calcium via calmodulin in nNOS and eNOS is done by using an increase in intracellular calcium ion (Ca^2+^). As calmodulin affinity to NOS is increased, electrons are transported from NADPH to the heme in the domain of oxygenase in the reductase domain. Because of the presence of various amino acids in the composition of the iNOS, calmodulin binds to very low Ca^2+^ intracellular concentrations around 40 nM (15–16).

The ovary is an organ that during reproductive cycles undergoes substantial structural and functional changes (17). Luteolysis is the structural degradation and function of the luteum in this period that suggests a decline in cell function. Analysis of corpus luteum in healthy ovaries is accompanied by increased production of reactive oxygen species (ROS) such as O_2_ and hydrogen peroxide (H_2_O_2_). Lipid peroxidation in the plasma membrane of the corpus luteum has been one of the effects of free radical development in ovarian tissue, which can contribute to the loss of gonadotropin receptors, decrease the formation of adenylyl cyclase-cyclic, cyclic adenosine monophosphate (cAMP) and ultimately decrease the steroidisation of the corpus luteum after its destruction (18).

Earlier studies have shown that NO plays an essential role to preserve the physiological balance of organs including ovaries (19). Motta et al. (20) have reported a direct relation between corpus luteum destruction and ovarian prostaglandin-F2α increase. They stated that depreciation of corpus luteum is directly linked to the reduced production of ovarian glutathione. Also, their study show that increased NO could increase the oxidising effect of the oxidase substances by the process of ovarian glutathione depletion, leading to the corpus luteum destruction (21). Another research from the same group showed the effect of NO on the ovary and found the use of L-NG-nitro-arginine methyl ester hydrochloride (L-NAME) to prevent the development intracellular NO in the ovary as a result of increased lipid oxidation (21–22).

## NO and the Female Reproductive System

### Regulation of Oocyte Maturation and Quality

**Figure 1:**
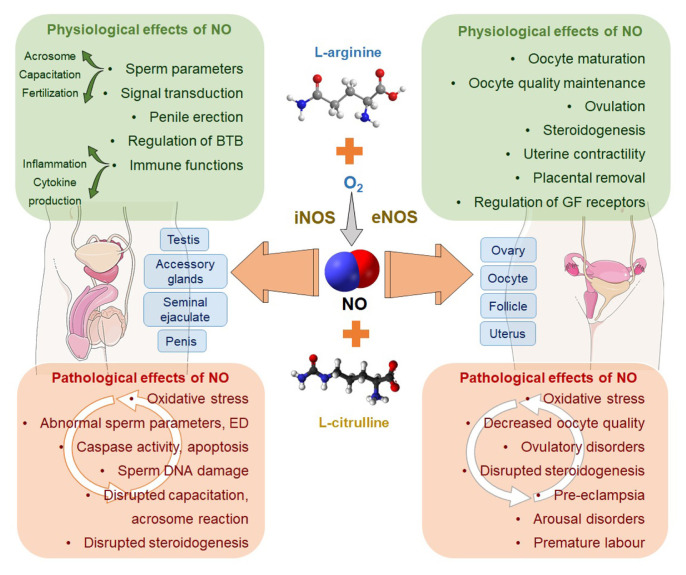
Physiological and pathological effects of NO on male and female reproductive functions Notes: BTB = blood-testis barrier; GF = growth factor; ED = erectile dysfunction

Full comprehension of the meiosis cell division has not yet been accomplished. However, more evidence indicates NO involvement in meiosis administration. NO has an important physiological role in oocyte maturation and fertilisation, embryo development and foetal implantation (23–25) (Figure 1). The presence of eNOS and iNOS in the mammalian oocyte has been confirmed during its follicular maturity. Inhibition in NO synthesis inhibition in the in vivo maturation of oocytes contributes to a decrease in blastocyst numbers and increases foetal apoptosis. High levels of NO often cause disruption of meiosis and the foetal growth of the cow, and a delay in meiosis restart (or resumption) (25). Goud et al. (26) have demonstrated that NO is a key factor in oocyte quality maintenance. An investigation of earlier literature reveals the binary role of NO in oocyte maturity. Moreover, Bu et al. (27) have described how the concentration dependence of NO has effects on oocyte maturity in the rat. For example, NO produced due to eNOS activity in the cumulus cells triggers oocyte maturity. Nonetheless, a higher NO concentration induces oocyte arrest by meiosis. A decrease in NO after a sudden increase in the development of luteinising hormone (LH surge) before ovulation may act as a key agent in resuming meiosis. Recent evidence has validated that the oocytes are able to block meiosis in the diplotene phase by expressing iNOS, which produces a sufficient amount of NO (28). Abbasi et al. (29) have reported that a cAMP cascade is the inducer platform of NO which starts meiosis in the rat oocyte, though cyclic guanosine monophosphate cascades support the inhibitory effort of NO.

### Follicular Development and Maturation

Pituitary gonadotropin is a crucial regulator for the final steps of follicular development and current data emphasise the balance in normal follicular growth between autocrine and/or paracrine influences. In many animal species, no presence of follicle fluid has been shown. The expression of NOS in follicles shows an inner ovarian system for synthesising NO and managing follicular growth. Several ovarian cell forms, as well as ovarian arteries, synthesise NO. In the tissue remodeling process, the ovarian macrophages kill apoptotic cells, in contrast to external macrophages, which is another NO source in ovarian tissue (6). In the phagocytosis of atretic granulosa and apoptotic luteal cells, these macrophage-rich regions of the ovary, such as theca sheet, corpus luteum and atretic follicles, are involved (25, 30).

### Ovarian Functions

The iNOS and eNOS have several roles in the ovulation process (6). With follicle development, NO synthesis increases and this increase is linked to increased estrogen. Similar changes in the amount of NO circulating in women undergoing in vitro fertilisation occur with the development of follicles. NO is continuously treated with the gonadotropin-releasing hormone and human chorionic gonadotropic hormone, and other hormones, such as the luteinising hormone, the follicle-stimulating hormone and progesterone have been observed (5). The intraperitoneal use of NOS inhibitors prevents ovulation in rats, which reflects the role of NO in ovulation (25, 30). Ovary stimulation by gonadotropins enhances the expression of both iNOS and eNOS genes, indicating that both NO isoforms play a part in the process of ovulation. The use of special n-methyl-l-arginine and aminoguanidine blockers to inhibit iNOS leading to a dose-dependent inhibition of ovulation in rats, which demonstrates the role of iNOS in ovulation (30). During follicular growth, eNOS is expressed in theca cells and in the granulosa cells of the follicle wall, and after ovulation, eNOS is expressed in the yellow body. The iNOS expression occurs in the theca cells and stroma cells in the immature ovary and during follicular growth, and iNOS is expressed in the outer layers of the yellow body after ovulation. Estimating the amount of iNOS means that the concentration of iNOS during follicular growth does not change, unlike eNOS (6). Recently, it has also been shown in mice models that NOS is involved in pubertal follicular maturation via the PI3K/AKT/FoxO3a pathway and mediate the mechanisms of follicular autophagia and apoptosis (6).

Since steroidogenesis is involved with theca cells, luteal granulosa cells and yellow body cells, it can be inferred that NO also plays a role in regulating steroid synthesis. However, iNOS expression in most organs of the body occurs solely in response to immune activation during any infection or trauma, and the physiological relationship of iNOS expression in the natural ovaries is still unclear at all levels. The decrease in expression or non-expression may be mainly due to the presence of macrophages and interleukin-1β in the ovary, and it is also possible that NO derived from iNOS acts as a support molecule for monitoring/growth. Interleukin-1β is a stimulant with NO synthesis in the fallopian tubes of humans and cows (6). On the other side, glucose rises in the middle of the menstrual cycle and glucose induces NO synthesis. It is therefore possible for NO and glucose to interact as follicles or inducers and promote pathways of follicular development (6). Sugino et al. (31) have reported in comparison to large and moderate-sized follicles a relationship between the concentration of NO in follicular fluid and apoptosis, where small follicles show more apoptosis. The concentration of NO (nitrite/nitrate), arginine and citrulline in these follicles is, however, not different. In addition, in human follicular fluid, the concentration of NO is increased, and this increase is directly related to the number of follicles and the concentration of estradiol. Taken together, these findings indicate that follicular growth is caused by the local development of NO and inhibits apoptosis (32).

### The Oviduct (Fallopian Tube)

In the uterine tube, the first evidence of the involvement of NO in the control of the functions of the uterine tube was the increased contraction due to endothelin in the presence of L-NAME, a NO synthesis inhibitor (5). By using prostaglandins, prostacyclin and cAMP, NO regulates the contractile movements in the human fallopian tube, so it also prevents tubal ectopic pregnancy (33). The presence of eNOS in the epithelial cells of the uterine tube is confirmed by multiple studies demonstrating the presence of calcium-dependent, as well as NOS-dependent, calcium-shaped types in the rat, cow, and human fallopian tube and immunohistochemical studies (34–35). The distribution of calcium-dependent NOS in the isthmus, fimbriae and ampulla of the uterine tube is the same, although the activity of NOS in the uterus during the proestrus period is comparatively lower than in the other stages of the estrus cycle (35). The release of NO appears to be able to improve sperm motility and protect the egg and sperm from damage caused by free oxygen radicals (6). NO can also influence the movements of the uterine tube’s epidermal cells. Some growth factor receptors, such as the epidermal growth factor, binding proteins and integrin, have been shown to be regulated by NO (36). In comparison to the physiological state, under certain pathological conditions, such as infection or endometriosis, NO synthesis in the uterine tube can be increased, leading to decreased fertility through a destructive or toxic effect on sperm cells as well as oocytes. In addition, the increased development of NO will influence the movements of the cilia and therefore the transfer of the embryo, and miscarriage can be the final outcome (6).

### Regulation of Uterine Functions

NO regulates the contraction during pregnancy of smooth muscle cells and uterus dilation; therefore, the role of NO has been considered to be critical in regulating pathophysiology and uterine biology (37). In the glandular epithelium, endometrial stromal cells, smooth muscle cells and mast cells, the presence of NOS indicates the involvement of NO in regulating uterine functions. In addition, for the control of myometrial function, such as contraction and relaxation of the uterus, local NO synthesis in the uterus may be essential (38). Although eNOS is expressed by smooth muscle cells (39), myometrium is one of the rare sections of uterine tissue that iNOS expresses with non-provocative effects (40), including i) promoting uterine traction to eliminate the residual uterine placenta after nitroglycerin delivery; ii) preventing preterm delivery and prolonging nitroglycerin pregnancy and iii) reducing the control of nitroglycerin pregnancy (6). A study conducted by Bansal et al. (41) have showed that in preterm labour, the expression of iNOS rather than eNOS and nNOS in humans was the strongest. Increases in NOS activity during pregnancy due to positive cytokine regulation and subsequent decreases during childbirth may be closely linked to inhibitory cytokines (42). The mouse uterus has been shown to interact with cyclooxygenase, NO and cytokine, and these factors can regulate the role of the uterus during pregnancy (43). In the uterus, ovarian hormones also induce iNOS expression and can control uterine function. The function of eNOS, however, is still unclear in epithelial cells and endometrial stroma. But it is likely that continuous NO development promotes processes such as menstruation and implantation by prostaglandin synthesis and by binding proteins. A NO derivative of eNOS resulting from the activation of guanylyl cyclase solution development or the decomposition of cyclooxygenase acts as an endometrial platelet aggregation inhibitor (6). Buhimschi et al. (40) have showed that both three NOS isoforms are expressed in the cervix. In addition, during natural and preterm labour in the cervix, the expression of iNOS rises and decreases in the uterus, and during childbirth in the cervix, nNOS, which is not expressed in the uterus during pregnancy, increases. There is no major shift in the expression of the eNOS gene during childbirth, irrespective of the involvement of iNOS and nNOS. These results suggest that NOS activity has a different purpose and concentration during childbirth in the uterus and cervix and can play a role in the reconstruction of connective tissue during the preparation of the cavity. During pregnancy and childbirth, the physiological and biological importance of NO indicates that the NO synthesis inhibitor (meaning L-NAME) prolongs the delivery time and therefore decreases the opening of the uterus (external os) and vagina external orifices (40, 44).

### Pregnancy, Placenta and Pre-eclampsia

Both eNOS and iNOS genes occur in the placenta, and in patients whose pre-eclampsia is elevated, eNOS expression in foetal and placental vessels occurs (45). Placental vessels are more important in the pathophysiology of pre-eclampsia, and it seems eNOS is important in conditions such as preeclampsia. During normal pregnancy, NO biosynthesis increases as gestation progresses and pre-eclampsia decreases, and eNOS is expressed in human placental syncytiotrophoblasts and extravillous trophoblasts (43, 45). In fact, in patients with pre-eclampsia who have an adaptive response to low perfusion and hypoxia, it may increase eNOS gene expression in the foetal and placental vessels. A study by Buhimschi et al. (40) has shown that administration of L-NAME to pregnant mice leads to a condition similar to pre-eclampsia.

## Nitric Oxide and the Male Reproductive System

### Testicular Functions

In the testicular vasculature endothelium, NO has been identified and its mechanism of action has been partially defined. Accordingly, NO can be shown to be effective in testicular perfusion, gonadotropin activation and its migratory pathway to the Leydig cells and to affect androgen displacement (38). NO is involved in the regulation of cell permeability, blood flow and myofibroblast contractile function in the testes, as well as in the regulation of steroid synthesis. It also controls sperm motility, as in conditions of low concentrations, NO increases sperm motility and when in moderate to high concentrations, it decreases sperm motility. A strong association between the concentration of NO and the percentage of sperm without motility was seen in human semen (seminal fluid) (46). NO is formed in a limited quantity under physiological conditions and allows the free radicals to scavenge. In contrast, under pathological conditions, such as infection, varicocele or diabetes mellitus, excessive NO production can cause sperm toxicity and also decrease sperm motility through peroxynitrite formation (47–48). The sperm ejaculated into the female reproductive tract is likely to lead to an immune response that causes iNOS activity and generates a significant amount of NO, which can lead to sperm non-maturation and ability (46). Endogenous NO inhibitor in seminal plasma can inhibit the activity of NOS and help to maintain low concentrations of seminal NO (which is beneficial to sperm functions rather being deleterious), to prevent Leydig cell injuries and to suppress sperm hypermotility associated with the process of capacity building (49).

### Penile Erection

In humans, NOS activity in the pelvic mesh, cavernous sinus nerves in penile tissue, dorsal branches of the penis, and deep arteries of the sinus cavernous is observed (6). This NOS activity indicates that NO is a physiological facilitator of erectile function in the rat penile neurons (which autonomically innervated the corpus cavernosum and penetrated the gland cavernous tissue) and in the neural network in the adventitia layer of the penile vessels. In the penile endothelium and the corpus cavernosum of the endothelium sinusoidum, eNOS is abundantly distributed in addition to expression in the nerves (39). Different results suggest that in smooth muscle cells of the cavernous sinus of the penis, all three iNOS, nNOS and eNOS genes are expressed. Administration of anti-androgenic medicine to healthy rats decreases nNOS and eNOS gene expression and reduces erection (50). Napoli and Ignarro (51) have also showed that the electrical stimulation of the isolated cell line into the intestine by the rabbit’s corpus cavernosum secretes NO. As per the available reports, it has been suggested that the erection is caused by NO and happens when a non-adrenergic-non-cholinergic neurotransmitter is reacted. Furthermore, direct L-NAME injection into the periventric nuclei results in apomorphine and oxytocin inhibition (as an erection inducer) (6).

## Nitric Oxide and Infertility

### Male Infertility

As discussed above, NO is an essential mediator of sperm functions (52–53). But besides its positive impact to mediate the important sperm functions, when its level exceeds the physiological value, it acts as a pro-inflammatory mediator in response to various infections, and thereby turns detrimental to sperm functions, especially impacting motility (54) and viability (55). Male infertility mainly owes to defective sperm functions and the sperm membranes are highly susceptible to lipid peroxidation as it is rich in polyunsaturated fatty acids (56–57). High levels of NO, being a potent nitrogen-derived free radical, may induce lipid peroxidation in sperm membrane, and hence impairing sperm intracellular microstructure, affected sperm chromatin integrity and curbing sperm functions (58–60) (Figure 1). However, despite considerable progress in research investigating the role of ROS in male infertility, the deleterious effects of reactive nitrogen species upon male reproductive functions are way under-researched.

Though evidence favours the physiological role of NO in mediating normal male reproductive functions, excess NO production reportedly have adverse effects upon semen parameters (54, 61–66) (Table 1). Researchers found that in the presence of 10^−4^ mol sodium nitroprusside, the number of sperm bound to the zona pellucida was much lower than in the control group that was not treated with NO. The viability of sperm was significantly reduced at the same sodium nitroprusside concentration. However, a few studies have found that NO has no influence on this sperm parameter (64, 67). Studies have also reported that toxic levels of NO can alter usual sperm appearance or morphology (55). Similarly to viability, a few investigations found that NO had no influence on sperm morphology. (63, 67). Furthermore, when compared to the seminal plasma NO concentrations (3.88 ± 0.53 μmol/L) of normal, fertile men, greater NO concentrations (5.74 ± 1.01 μmol/L) in infertile men are more likely to result in capacitation inhibition. Furthermore, increased NO levels were connected to a reduction in sperm metabolism in these infertile men (68). In general, to prevent adverse effects of NO on sperm functions, it is vital that body’s natural defense appropriately manage the levels of NO.

NO toxicity has been linked to leukocytospermia, which is characterised by oxidative stress (OS) and an excess of white blood cells in the seminal ejaculate (1 × 10^6^ WBC/mL) (69–71). Leukocytospermia interferes with sperm motility and results in spermatozoa agglutination, which can contribute to infertility (64). It also causes the production of cytotoxic cytokines, which might be a sign of an underlying infectious disease condition (72). Leukocytospermia is suggested as the major causative of OS and the most obvious mechanism that result in excessive NO production (64).

Varicocele, which is one of the most prevalent causes of male infertility, has been also connected with high NO levels. Varicocele is a disorder that swells and widens the veins of the pampiniform plexus across the cord restricting blood circulation in this location (73–74). The numerous varicocele-associated symptoms, including testicular hypoxia, germ cell dysfunction due to small vessel occlusion and an increase in scrotal temperature, decrease in gonadotropin production and testicular dysfunction, have been reported to be involved with NO, specifically produced by iNOS. Despite these potential varicocele mediated disruptions, the actual mechanism by which NO plays a role in its pathophysiology, remains unveiled (73–75).

The mechanism of erectile dysfunction (ED) is also influenced by NO. The inability to develop or sustain erections adequate for a satisfactory sexual intercourse is known as ED (76–77). As previously stated, erection is mediated via the soluble guanyl cyclase/cGMP/cGMP pathway, which is stimulated by NO. This process involves the phosphorylation of a number of proteins, which results in smooth muscle relaxation and blood filling of the sinusoidal spaces of penis. However, as NO competes with oxyhemoglobin or superoxide anion to produce harmful peroxynitrite in ED, there may not be enough NO to activate this pathway (76–78).

### Female Infertility

NO is an autocrine and paracrine modulator of ovarian functions, mainly folliculogenesis. When present at low concentrations (< 1 μmol/L), NO acts via the activation of soluble guanyl cyclase and cyclic guanosine monophosphate (cGMP) (79–80). Evidence shows that follicular NO concentration is elevated during the secretory phase of the menstrual cycle while reach its peak during the mid-cycle (81). When follicular fluid NO concentration is low, the follicles possess mature oocytes that can become fertilised (82). However, with increase in concentration of follicular fluid NO, it has been shown that there is diminished embryo quality and rate of cleavage. It has also been reported that serum NO concentrations were increased in infertile women with tubal factor- or peritoneal factor-infertility (83). When follicular fluid NO concentrations exceed physiological limit, it may lead to implantation failure and is associated with lower pregnancy rates. In vitro studies showed that NO may even trigger uncontrolled apoptosis of the embryo cells and fragmentation of the embryo (84).

## Conclusion and Future Perspectives

NO is a short-term lipophilic molecule and is synthesised in the body by a variety of species. It has also been incorporated into many physiological cascades as an intracellular messenger. Increasing the blood flow in sexual organs, vascular tonic control, genital tract formations and defense mechanisms are, among other things, NO associated with cell growth, apoptosis, reproductive signal transduction. NO reacts to proteins, thiol groups and oxygen-active species. It is able to defend or poison cells due to its concentration and place of action. It is a nitrogen-active species and is a member of the vasculogenesis and angiogenesis, development and puberty, senescence and apoptosis in the majority of physiological methods. Synthesised by NOS, NO plays a major role for the male physiological system as well as for sperm motility, maturity, quality and ability, and oocyte binding to sperm in physiological waterfalls such as erectile functions and androgen secretions. In addition, this supposedly simple molecule is involved in other roles, such as the evolution of germ cells, the links between Sertoli cells and germ cells in blood-testis barrier, homodynamic contraction and apoptosis of germ cells. In addition, because of its widespread distribution in both normal and diseased testis tissue, NO is regarded as a key factor in male fertility. In the natural or pathological state, the levels of expression of eNOS and iNOS are different, and overexpression of these two isoforms is a possible cause of destructive fertility processes, including low sperm motility and viability, destruction of testis tissue, activation of apoptosis in germ cells, and, literally, spermatogenesis disturbance. In manufacturing ovarian steroids, ovulation and follicular apoptosis, NO is a significant factor. In the ovulation process, iNOS is the major isoform. In other words, increased iNOS activity contributes to an increased quantity of NO, which induces prostaglandin production and causes inflammatory cascades that can cause follicular rupture and atresia. In the luteal and granulosa cells, NO concentration elevation prevents steroid synthesis. Overproduction of NO in the uterus results in toxicity and inflammation in epithelial cells and immune rejection of implantation, as NO is a significant paracrine mediator of different biological processes and plays a key role in both the reproductive cycle and embryo implantation.

At present, the number of infertile couples has substantially increased, with various categories of patients include lower fertilisation rates, increased abortion levels and high morbidity. One of the key chemical and pathophysiological influences in this respect is an increased degree of NO. The scientific community requires new technologies and synthetic materials to calculate, identify and monitor its level, due to various roles of NO, and various functions in the molecular signaling of the reproductive system for men and women. In both pathological and physiological processes, the paradoxical function of NO depends on the body’s overall state and on the oxidant/antioxidant balance mechanism.

## Figures and Tables

**Table 1:** NO and sperm parameters

Researchers	Concentration NO/NOS/NO producer	Effects on sperm parameters
Balercia et al. (54)	0 nmol–3 nmol × 10^6^ NO	NO levels were found to be inversely related to sperm motility (*P* < 0.0007). Following cryopreservation, NO released by sodium nitroprusside, plays a significant role in keeping the sperms viable.
Rosselli et al. (61)	50 nmol–100 nmol NO	NO improves post-thaw sperm viability at lower concentrations.
O’Bryan et al. (62)	50 nmol–100 nmol eNOS	Aberrant patterns of sperm eNOS expression associated with decreased sperm motility (*r* = −0.46; *P* < 0.05). NO improves post-thaw sperm viability at lower concentrations.
Miraglia et al. (63)	S-nitrosoglutathione (100 nmol/L), 2-phenyl-4,4,5,5-tetramethylimidazoline-3-oxide-1-oxyl (100 μmol/L), 1H-[1,2,4]oxadiazolo[4,3-a]quinoxalin-1-one (50 μmol/L), 8-bromoguanosine-3′, 5′-cyclic monophosphate (1 μmol/L), 8-bromoguanosine-3′, 5′-cyclic monophosphorothioate, Rp-isomer (10 μmol/L)	Progressive motility of human sperm did not change following a 20-min incubation with all these NO releasing agents (*P* < 0.05).
Tomlinson et al. (64)	10^−6^ to 10^−4^ mol sodium nitroprusside	Sodium nitroprusside significantly reduced the progressive motility, percentage and concentration of motile sperms in all doses (*P* < 0.005). Sperm viability did not differ significantly from that of control sperm (*P* > 0.05). Sperm viability has been decreased following NO treatment.
Bolaños et al. (65)	Sodium nitroprusside (0.25 μmol–2.5 μmol) S-nitroso-N-acetylpenicillamine (0.012 μmol–0.6 μmol)	Sperm viability (*P* < 0.05) and motility (*r* = 0.740; *P* < 0.01) have been decreased following NO treatment.
Archer (66)	S-nitroso-N-acetylpenicillamine 0 nmol–1.2 nmol/10^6^ spermatozoa	Percentage of immotile spermatozoa were found to increase with A higher concentrations of NO (*P* < 0.01).
